# Design, analysis, and reporting of pilot studies in HIV: a systematic review and methodological study

**DOI:** 10.1186/s40814-021-00934-9

**Published:** 2021-11-30

**Authors:** Hussein Ali El-Khechen, Mohammed Inam Ullah Khan, Selvin Leenus, Oluwatobi Olaiya, Zoha Durrani, Zaryan Masood, Alvin Leenus, Shakib Akhter, Lawrence Mbuagbaw

**Affiliations:** 1 Department of Health Research Methods, Evidence, and Impact, McMaster University, 1280 Main Street West, Hamilton, Ontario L8S 4L8 Canada; 2 Biostatistics Unit, Father Sean O’Sullivan Research Centre, St Joseph’s Healthcare, Hamilton, Ontario Canada; 3 Faculty of Health Sciences, McMaster University, Hamilton, Ontario Canada; 4 Michael G. DeGroote School of Medicine, McMaster University, Hamilton, Ontario Canada; 5 Joan C. Edwards School of Medicine, Marshall University, Huntington, WV USA; 6 Department of Kinesiology, Faculty of Science, McMaster University, Hamilton, Ontario Canada; 7 Center for the Development of Best Practices in Health, Yaoundé, Cameroon

**Keywords:** Pilot study, Feasibility study, Feasibility outcome, Progression criteria, Key population, HIV

## Abstract

**Background:**

Pilot studies are essential in determining if a larger study is feasible. This is especially true when targeting populations that experience stigma and may be difficult to include in research, such as people with HIV. We sought to describe how pilot studies have been used to inform HIV clinical trials.

**Methods:**

We conducted a methodological study of pilot studies of interventions in people living with HIV published until November 25, 2020, using Medline, Embase, and Cochrane Controlled Register of Trials (CENTRAL). We extracted data on their nomenclature, primary objective, use of progression criteria, sample size, use of qualitative methods, and other contextual information (region, income, level, type of intervention, study design).

**Results:**

Our search retrieved 10,597 studies, of which 248 were eligible. The number of pilot studies increased steadily over time. We found that 179 studies (72.2%) used the terms “pilot” or “feasibility” in their title, 65.3% tested feasibility as a primary objective, only 2% used progression criteria, 23.9% provided a sample size estimation and only 30.2% used qualitative methods.

**Conclusions:**

Pilot studies are increasingly being used to inform HIV research. However, the titles and objectives are not always consistent with piloting. The design and reporting of pilot studies in HIV could be improved.

**Supplementary Information:**

The online version contains supplementary material available at 10.1186/s40814-021-00934-9.

## Introduction

Wastefulness in medical research is a major concern for researchers and funders and has been estimated to be at 85% of research investment [1]. There are several contributors to this waste. These include researchers not asking relevant questions, study results being inaccurately reported, and the inappropriate use of study design [2]. Recent work has demonstrated that pilot studies are very effective in reducing waste [3]. It was found that by employing pilot studies, the UK’s National Institute for Health Research’s (NIHR) Research for Patient Benefit (RfPB) Program saved approximately £20m, as otherwise non-feasible studies would have been conducted [3]. Pilot studies are especially useful in fields where participants are difficult to recruit and retain. However, despite the recognized value of pilot studies, there is still considerable confusion surrounding what constitutes a pilot study, how they should be designed, and how researchers decide whether they should proceed with the full study [4].

A pilot study is often described as a scaled down version of a larger study with feasibility, the assessment of the ability to conduct the full-scale study, as the primary goal [5]. Given the numerous challenges of recruiting and retaining participants in HIV research, pilot studies are particularly important in this field [6–15]. In this context, pilot studies could help researchers understand how they can adjust their procedures and reduce waste, especially when working with populations that experience difficulties, including stigma and discrimination [3]. People with HIV may belong to other key populations and face additional social stigma (e.g., men who have sex with men [MSM], commercial sex workers [CSWs], or people who inject drugs [PWID]), making participation in research more difficult [8–12, 16]. Studies have found that certain subpopulations (e.g., women, PWID, and African, Caribbean and Black (ACB) peoples) have dropout rates in research studies ranging from 30 to 50% [17–19].

The primary goal of this study was to describe the design, analyses, and reporting of pilot studies in HIV. The main outcomes of interest were the following:

1. The nomenclature of pilot studies (“pilot” or “feasibility” in the title)
2. Their declared primary objective (feasibility or effectiveness) and primary outcome
3. The use of progression criteria (criteria that would inform the decision to move to a larger trial)
4. Sample size estimation or justification (a description of why the sample size was chosen)
5. Use of qualitative methods (inclusion of qualitative assessments to inform feasibility)
6. The inclusion of key populations as defined by United Nations Program on HIV/AIDS (UNAIDS) [20–23]

Outcomes 1-5 have been identified as common shortcomings in pilot studies [6–15, 24–35]. The inclusion of key populations would indicate that a more diverse sample of people with HIV was included.

## Methods

We conducted a methodological study of pilot studies in the HIV literature as per the guidelines reported by Murad and Wang for reporting meta-epidemiological research [36].

### Ethics

This study used publicly available secondary data and therefore ethics review was not required.

### Criteria for inclusion

All pilot studies of interventions conducted exclusively in people with HIV and published in English were eligible. We included randomized and non-randomized studies, using mixed or quantitative methods with at least one feasibility outcome [4]. Outcomes were deemed to be feasibility outcomes if they fit into a category outlined by Thabane et al. [4]. These categories include (1) assessing the processes involved in the study, (2) evaluation of resources required for the study, (3) management of potential human and data management problems, and (4) assessment of intervention safety, dose response, and variance of effect.

### Search method for identifying pilot studies

We conducted an exhaustive search of the following databases: Medline, Embase, and the Cochrane Central Register of Controlled Trials (CENTRAL). These databases were searched from inception to November 25, 2020. Our search strategy was developed in collaboration with a librarian at the library services of the McMaster Health Sciences Central Library. The key concepts included in the search were “pilot,” “feasibility,” “proof-of-concept,” “exploratory,” “preliminary,” and “HIV.” The complete search strategies are included in the supplementary materials.

### Screening and data extraction

We compiled the references and removed duplicate citations using Endnote X9 reference manager software [37]. We screened the remaining references first by their title and abstract and then by examining their electronic full texts. Both screening steps were done in duplicate by two independent reviewers using the Covidence data management platform for systematic reviews provided by McMaster University [38]. The reviewers attempted to resolve discrepancies by discussion and included a third reviewer if consensus could not be reached.

Data from included references were extracted using a piloted data-extraction form on RedCap [39]. Basic bibliometric information extracted from the studies included the following: the first author’s last name, study title, year of publication, journal of publication, and country of study (both region and income level). Region was determined using the regional groupings definitions provided by the World Health Organization (WHO), and income level was determined as per the World Bank Criteria [40, 41]. Other information collected included presence of a feasibility/pilot-identifying term in the title, study objectives, whether feasibility was a primary outcome, the use of progression criteria, a sample size justification, qualitative methods and study design, intervention type, and the inclusion of key populations (as defined by the UNAIDS and WHO [20–23]); key populations for which data were collected included (1) PWID, (2) MSM, (3) incarcerated populations, (4) CSW, (5) pregnant women, (6) children, (7) youth, (8) indigenous people, (9) ACB people, (10) women, and (11) transgender people. Progression criteria, having a feasibility related primary outcome and key labeling of pilot study status, are key characteristics of a pilot study. This information was extracted as these study characteristics have been found to impact study outcomes [42, 43]. We contacted authors via email to clear up ambiguity or to collect missing data.

### Analysis

We conducted a descriptive analysis and reported counts and percentages for categorical variables and median (minimum, maximum) for continuous variables.

## Results

### Results of search

**Fig. 1:**
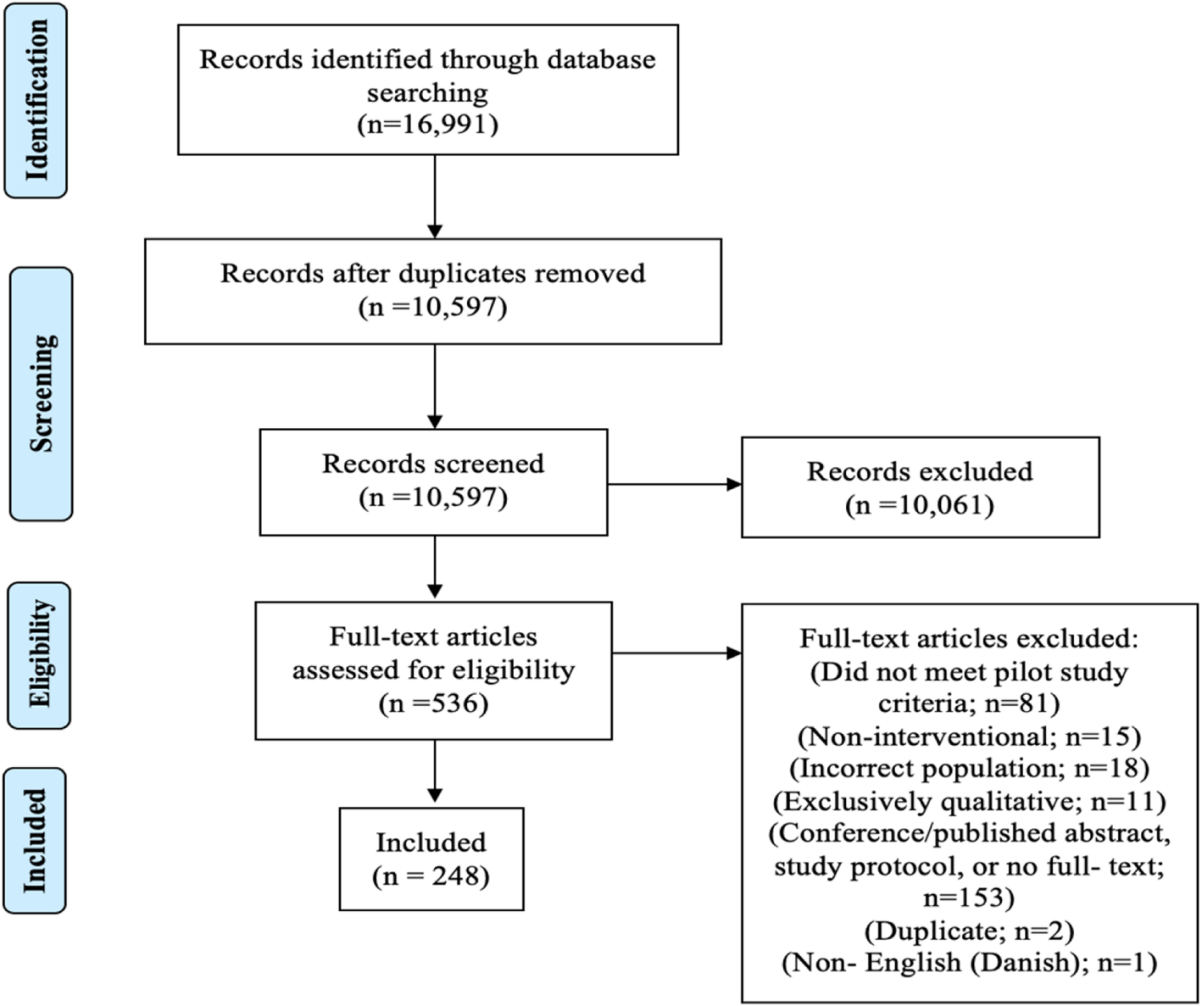
Flow chart of study screening and selection

Our search returned 10,597 articles for title and abstract review. Of these, 536 were retrieved for full-text review. Only 248 articles met our eligibility criteria. Figure 1 is a flow chart of our screening and selection procedures.

### Characteristics of included studies

**Fig. 2:**
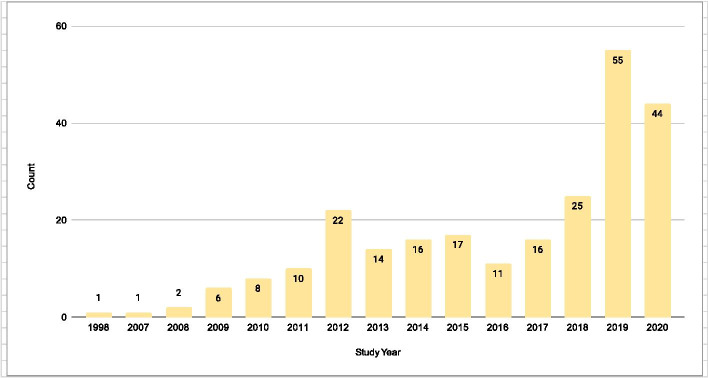
Yearly trends in publication of pilot studies in the HIV literature

The 248 studies included in our review were published between 1998 and 2020, with a steady increase over time (Fig. 2). Less than half of the included studies were randomized (108/43.5%). The majority investigated a pharmaceutical intervention (227/91.5%); were conducted in the Americas (137/55.2%) or Africa (71/28.6%). Further characteristics are reported in Table 1.

**Table 1:** Baseline characteristics of included studies

Characteristics	Statistic (***n*** = 248)
**Design,** ***n*** **(%)**	
Randomized control trial	108 (43.5)
Non-randomized trial	140 (56.5)
**Intervention type,** ***n*** **(%)**	
Pharmaceutical	21 (8.5)
Non-pharmaceutical	227 (91.5)
**Number of study sites,** ***n*** **(%)**	
Single site	143 (57.7)
Multiple sites	105 (42.3)
**Region,** ***n*** **(%)**	
Africa	71 (28.6)
Americas	137 (55.2)
Southeast Asia	11 (4.4)
Europe	16 (6.5)
Western Pacific	8 (3.2)
Mixed region	5 (2)
**Country income level,** ***n*** **(%)**^**a**^	
High income	151 (60.9)
Upper-middle income	37 (14.9)
Lower-middle income	25 (10.1)
Low income	33 (13.3)
Mixed income	2 (0.8)
**Qualitative methods,** ***n*** **(%)**	75 (30.2)
**Sample size: median (min, max)**	40 (3, 8794)
**Key population,** ***n*** **(%)**	
Contain any key population	134 (54)
*Specific key populations, n* (%)	
Injection drug User	4 (1.6)
MSM	20 (8.1)
Incarcerated populations	0 (0)
Commercial sex workers	1 (0.4)
Pregnant women	11 (4.4)
Children	5 (2)
Youth	28 (11.3)
Indigenous	0 (0)
African, Caribbean, Black	77 (31)
Women	24 (9.1)
Transgender	3 (1.2)
**Progression criteria prespecified,** ***n*** **(%)**	5 (2)
**Trial outcome,** ***n*** **(%)**	
Proceed to larger study	65 (26.2)
Do not proceed	183 (73.8)

### Outcomes

#### Nomenclature

This has been clarified. New sentence: “Most studies were easily identifiable as pilot or feasibility studies, with 179 studies (72.2%) including the terms pilot, feasibility, or a feasibility outcome in the study title to denote the pilot status.” Feasibility outcomes were used to denote pilot status in 27 of these studies (15.1%). This was often done with the feasibility outcome “acceptance.” For example, a study was titled “Acceptability of a trial of vaginal progesterone for the prevention of preterm birth among HIV-infected women in Lusaka, Zambia: A mixed methods study” [44]. The remaining 69 studies (27.8%) had no indication of their pilot nature in the title.

#### Objectives

Study feasibility objectives were often clearly stated in the beginning or at the conclusion of the introduction. The primary objective was feasibility in 162 studies (65.3%). The remaining studies had goals centered around informing the sample size of the larger study, assessing efficacy, intervention development and to assess the reliability of a measure, with feasibility treated as a secondary outcome.

#### Outcomes

Feasibility outcomes were used as a primary outcome in 157 (63.3%) studies. We found that the feasibility outcomes assessed in the studies in this review could be grouped into 11 categories in total (Table 2). The most common outcomes were acceptance and retention of participants (180/72.6%; 135/54.4%, respectively), as well as evaluating participant enrolment (106/74.6%) and compliance (131/52.8%) to the intervention and study procedures. Trialists also often sought feedback (90/36.3%), primarily from participants and occasionally from study staff. Miscellaneous feasibility outcomes were also assessed and defined in 41 studies. The most common of these were implementation (34/13.7%), intervention initiation (6/2.4%), and consent rate (1/0.4%). The same outcome was defined differently in most instances, see Table 2. The CONSORT extension for pilot studies distinguishes between primary and secondary feasibility outcomes. The former are outcomes that inform the decision about progressing to a full RCT. Secondary feasibility outcomes are those related to patient centered outcomes [45].

**Table 2:** Feasibility outcomes and definitions

Outcome^a^ (*n*/%)	Definition
Acceptance (180/72.6%)	a) Participant satisfaction with the intervention and study procedures (165/91.7%) b) Participant recruitment or enrolment rate (10/5.6%) c) Intervention completion (16/8.9%) d) Participant retention (11/6.1%) e) Participant feedback (4/2.2%) f) Intervention usability (2/1.1%)
Contamination (2/0.8%)	The proportion of participants that deviated from their allocated intervention and partook in the alternative intervention
Compliance^b^ (131/52.8%)	a) Participant attendance of any intervention sessions or simply adherence to assigned intervention (27/20.1%) b) The number of sessions attended and engagement with the intervention (110/82.1%)
Data completion (9/5%)	The proportion of data expected to be collected which was not
Enrolment (106/42.7%)	a) The proportion of eligible participants who consented to join the study (95/90.5%) b) The proportion of participants which were recruited and randomized (9/8.5%) c) The proportion of participants recruited, consented and were randomized (2/1.9%)
Feedback (90/36.3%)	What participants and study staff thought of the intervention, study procedures and their time in the trial
Fidelity (37/14.9%)	The ability of the trialists to adhere to study protocol
Randomization (11/4.4%)	The ability to successfully randomly allocate participants to the different arms in a trial
Retention (135/54.4%)	The proportion of participants which remained in the study till the primary endpoint (either end of the intervention or a set follow-up period)
Resources (16/6.5%)	An evaluation of the resources required to conduct the study
Timeliness of intervention (2/0.8%)	Assessment of the ability to administer the intervention in the prespecified time
Other (34/13.7%)	a) Implementation—the ability to deliver the intervention to participants (17/65.38%) b) Initiation—the proportion of eligible participants which were recruited, consented to join the study and actually began using or were administered the intervention (6/23.07%) c) Consent rate—the proportion of eligible patients which consented to joining the study (1/3.8%)

^a^Studies may possess several feasibility outcomes

^b^Participant acceptance was often defined and assessed in multiple ways in the same study

#### Samples size estimation/justification

A sample size estimation was provided in 59 studies (23.8%). Forty-two of these studies (71.1%) provided an appropriate justification. Sample size justifications could be grouped into 6 categories. The most common justification was the use of a conventional sample size calculation with the intervention effect size to calculate a suitable sample size (20/33.9%). Researchers also equally relied on similar studies (6/10.2%) and on recommendations in the literature (6/10.2%). Three (5.1%) studies determined their sample size based on the resources available to conduct the study. Two (3.4%) studies used a proportion of the sample size of the larger study to justify their estimation. Finally, miscellaneous justifications were provided in 5 (10.2%) studies. For example, Tsima et al. justified their sample size using their estimation of expected recruitment rate [46].

#### Progression criteria

Only 5 (2%) of the included studies mentioned the progression criteria for their pilot studies. These studies were all RCTs published in 2020. Of these 5 studies, 3 (60%) were conducted in African countries. Of the 3 conducted in African countries, 2 (40%) were in low-middle-income countries and the other (1/20%) conducted in an upper-middle-income country. The other two were conducted in Europe and America.

### Qualitative methods

Qualitative methods were included in only 75 studies (30.2%). Analysis was primarily conducted using data collected from participants. However, some studies also collected data from staff as well (25/33.33%).

### Key populations

Close to half of the studies included a key population (134/54.0%). The complete composition of the patient sample is found in Table 1. ACBs represented the largest subpopulation among studies including key populations at 57% (*n* = 77). Only 24 (17.9%) of studies sought to purposely recruit women. Youth (28/17.2%) and MSM (20/14.9%) made up the remaining prominent vulnerable populations present. Other vulnerable populations present were PWID, CSW, pregnant women, children, and transgender women.

## Discussion

To the best of our knowledge, this is the first methodological study of pilot studies in the HIV literature. We found that although pilot studies are becoming increasingly common in the HIV literature, there are considerable gaps in how they are labeled, designed, and how their findings are reported.

It is important to clearly label pilot studies and to use the correct terminology when doing so. Making pilot studies easier to identify helps inform readers that the primary goal of the study is to assess feasibility. We found that most authors (179/72.2%) labeled their studies clearly in the title.

Other reviews have found similar results. In two reviews of pilot studies in the cluster RCT literature and of pilot studies in the *Clinical Rehabilitation* journal, 83% and 87% of studies contained the terms pilot or feasibility in their title, respectively [24, 25]. The latter found that more than half of the studies used the pilot and feasibility terms interchangeably [25].

Our findings regarding authors replacing the primary feasibility objectives of their pilot studies, with other outcome(s) unrelated to feasibility, are in line with those of other authors [25–27]. The primary objective of a pilot or feasibility study must be to assess the feasibility of a larger study. As a result, assessing efficacy in a pilot study is inappropriate as this is not the primary goal of pilot studies and they are not powered to do so. However, smaller, non-pilot studies are important as they are important in hypothesis generation and in challenging widely held beliefs and common practices [47]. These studies should be encouraged and labeled properly as they also have a place in the literature [47]. The CONSORT extension for pilot studies of RCTs reinforces the requirement that pilot RCTs must have feasibility as their primary outcome by requiring feasibility outcomes be pre-specified and clearly defined [45].

Pilot studies must outline how authors intend to use their findings to inform future steps with pre-specified progression criteria [4]. However, only 5 (2%) studies assessed in this review included such criteria. Not reporting progression criteria is problematic, as we are unable to evaluate the criteria which the authors used to base their decision. A recent methodological review found that only 19.8% of studies included progression criteria [28]. Meanwhile, a review of cluster RCT pilot studies found that 89% of studies specified progression criteria [27]. However, the latter found that only 17% justified the criteria [27].

Currently, the CONSORT extension for pilot studies of RCTs requires reporting progression criteria, if applicable [45]. However, progression criteria are a key requirement of pilot studies, and this criterion should be strengthened to require the specification of the prespecified criteria. Progression criteria would improve the interpretability of the study [45]. The pilot studies assessed lacked both estimations and justifications for their sample sizes. Only 23.8% (*n* = 59) of studies had estimations for their sample size. However, even among these studies, 28.8% (*n* = 17) did not justify them. Chan et al. obtained similar results as they found that only 44% of pilot cluster RCTs justified for their sample size (27). As pilot studies do not aim to test hypotheses, formal power considerations are not necessary. However, it is still necessary to justify the sample size selected. As for now, guidance is only available for RCTs. As a result, concerns relating to progression criteria pertain more to pilot trials and are not always relevant for all pilot studies.

Several approaches can be used, such as targeting a percentage of the larger study’s sample size [48], having a set minimum number of participants per arm [48, 49], and using a stepped approach determined by standardized effect sizes [50]. Viechtbauer et al. have also proposed a sample size equation using the probability of a specific problem occurring during the trial to determine a sample size [51].

There have been recent calls for greater inclusion of qualitative methods in pilot studies [52, 53]. The use of qualitative methods has been shown to help refine study procedures, including optimizing recruitment and retention [29, 30]. By incorporating these methods in pilot studies, investigators are able to set realistic targets, craft pragmatic procedures, and ask and answer a wider range of questions, while gaining granular detail [53, 54]. As mentioned above, identification of pilot studies may be challenging given sub-optimal labeling. More so, identification of feasibility and pilot studies containing qualitative methods may be harder to identify as the qualitative components may be reported separately. These qualitative papers are harder to find if their titles do not contain the terms “pilot” or “feasibility.”

The HIV patient population is diverse and people with HIV, a stigmatized population, often belong to other stigmatized groups as well. This includes MSM, ACB, PWIDs, and CSWs, and is harder to recruit and retain in studies [6–15]. This impacts the ability to study these populations [6–18]. As a result, it is important to employ strategies that reduce barriers to participation and to evaluate them using pilot studies before committing to a full study [33, 55]. Few studies included these key populations, and this could potentially compromise feasibility in the larger study as the challenges in recruiting and retaining them have not been investigated in the pilot.

Our meta-epidemiological study has some weaknesses. Firstly, we were reliant on the authors’ conclusions to determine study feasibility and progression to the larger study. In addition, some of the studies included were evaluated using methodological advances developed after they were published, and this may explain why these approaches (sample size estimation, progression criteria etc.) were not used.

While our study does have weaknesses, it also has several strengths. Our review was robust as our search was highly sensitive, as demonstrated by the exceptionally large number of studies screened (*n* = 9297). The concepts searched, pilot studies and HIV, were purposefully broad as we are interested in all HIV interventions. In addition, with our study being specific to the HIV literature, we are able to evaluate a particular area of research where pilot studies will increasingly play a bigger role.

Future studies could expand on this work and evaluate the impact pilot studies have on the final study. Furthermore, future studies can assess the change in study quality since the introduction of the CONSORT extension for pilot studies.

## Conclusion

Pilot studies are increasingly being used in the HIV field. However, feasibility outcomes were not always the primary outcomes of the pilot studies evaluated. In addition, many key pilot study requirements, such as selecting a sample size, crafting progression criteria, and defining feasibility outcomes were often not included. Higher quality pilot studies are needed.

## Supplementary Information

**Additional file 1:.** Search Strategies.

## Data Availability

The datasets used and/or analyzed during the current study are available from the corresponding author on reasonable request.

## Abbreviations

NIHR: National Institute for Health Research
RfPB: Research for patient benefit
HIV: Human immunodeficiency virus
CENTRAL: Cochrane Central Register of Controlled Trials
WHO: World Health Organization
RCT: Randomized controlled trial
ART: Antiretroviral therapy
ACB: African, Caribbean, and Black
MSM: Men who have sex with men
PWID: People who inject drugs
CSW: Commercial sex workers
STROBE: STrengthening the Reporting of Observational studies in Epidemiology
NGT: Nominal group technique
