# Supplementary figures and images for: Design, analysis, and reporting of pilot studies in HIV: a systematic review and methodological study
Source: Pilot Feasibility Stud. 2021 Nov 30;7:211. doi: 10.1186/s40814-021-00934-9 (PMC8630899; doi:10.1186/s40814-021-00934-9)

**Search Strategies**

**Embase**


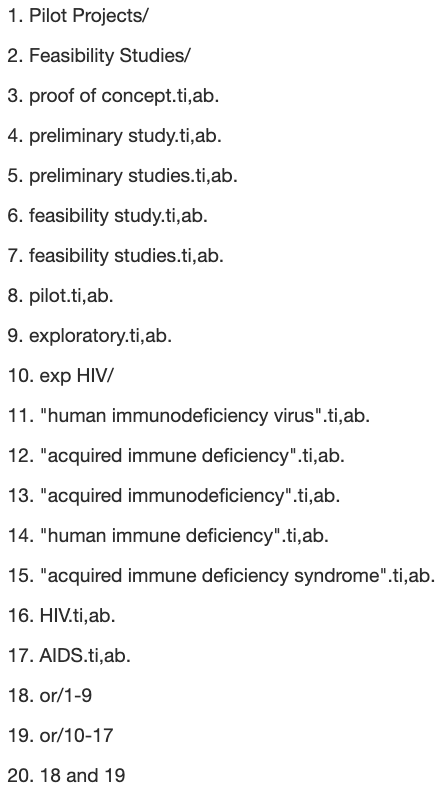


**Medline**


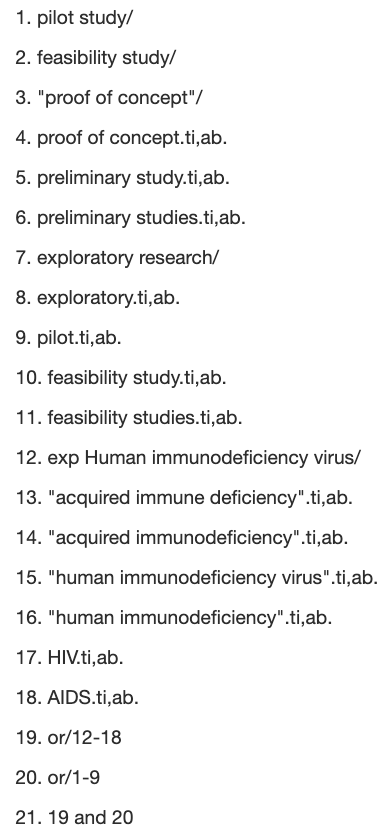


**CINHAL**

**
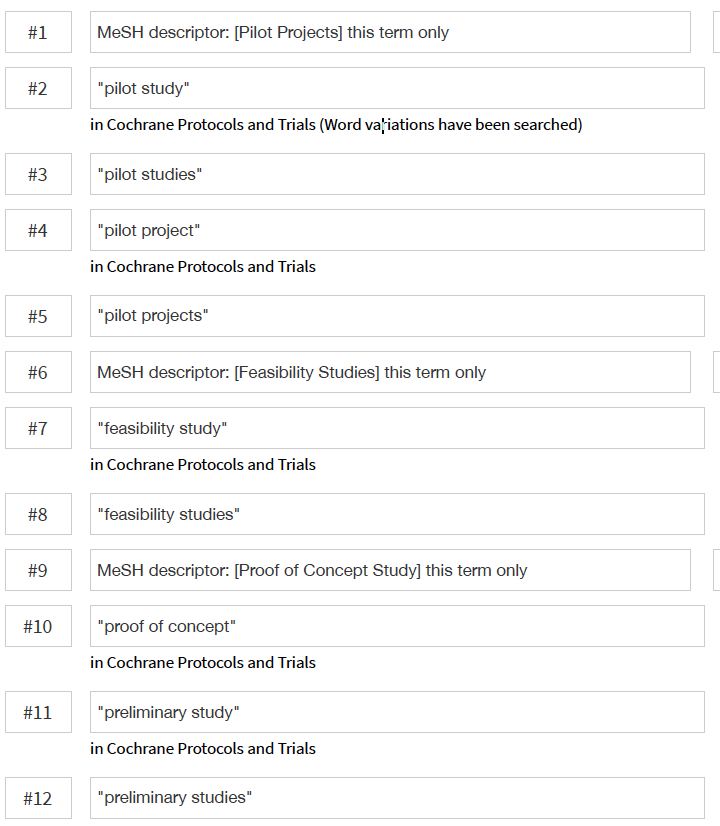
**

**
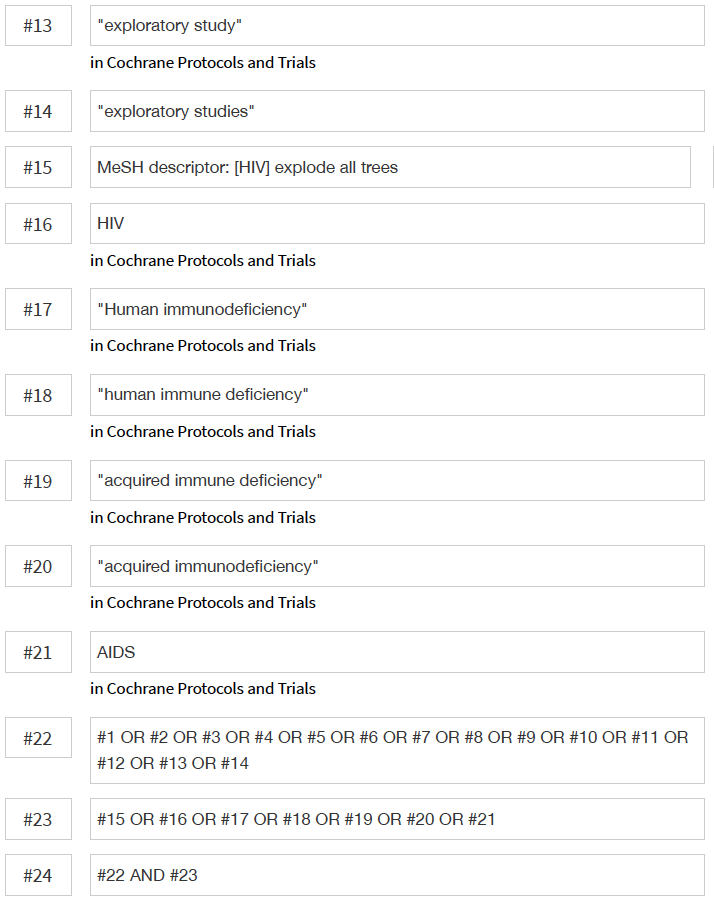
**

Supplement: Supplementary file 1 — Additional file 1:. Search Strategies. [file 40814_2021_934_MOESM1_ESM.docx]
